# Thrombophilic risk factors for retinal vein occlusion

**DOI:** 10.1038/s41598-019-55456-5

**Published:** 2019-12-12

**Authors:** Maria J. Vieira, António Campos, Anália do Carmo, Henrique Arruda, Joana Martins, João P. Sousa

**Affiliations:** 1 Ophthalmology Department, Centro Hospitalar de Leiria, Leiria, Portugal; 2 Coimbra Institute for Clinical and Biomedical Research (iCBR), Faculty of Medicine, University of Coimbra, Coimbra, Portugal; 3 ciTechCare, Center for Innovative Care and Health Technology, Instituto Politécnico de Leiria, Leiria, Portugal; 4 Clinical Pathology Department, Centro Hospitalar Universitário de Coimbra (CHUC), Coimbra, Portugal; 5 CNC.iCBR Consortium, University of Coimbra, Coimbra, Portugal; 6 Medical Sciences Department, Faculty of Health Sciences, University of Beira Interior, Covilhã, Portugal

**Keywords:** Biomarkers, Risk factors

## Abstract

The aim is to study risk factors for retinal vein occlusion (RVO), such as thrombophilic and cardiovascular risk factors (CRF). A retrospective consecutive case series of 60 patients with RVO was made, tested for CRF, hyperhomocysteinemia, lupic anticoagulant, antiphospholipid antibody and 5 gene variants: factor V (FV) Leiden (G1691A), factor II (PT G20210A), 5,1-methylenetetra-hydrofolate reductase (MTHFR; 677 C > T and 1298 A > C), plasminogen activator inhibitor 1 (PAI-1; 4 G/5 G). More than 1 CRF were present in 36 patients (60%), which had a significantly higher mean age at diagnosis (66.7 ± 12.9 *versus* 59.5 ± 13.7 with ≤1 CRF, [*t*(57) = −2.05, *p* = 0.045, *d* = 0.54). Patients with thermolabile MTHFR forms with decreased enzyme activity (T677T or C677T/A1298C) had a significant lower mean age [57.6 ± 15.1; t (58) = 3.32; p = 0.002; d = 0.846] than patients with normal MTHFR enzyme activity (68.5 ± 10.2). Regarding CRF and thermolabile forms of MTHFR, the mean age at diagnosis could be significantly predicted [F(2,56) = 7.18; *p* = 0.002] by the equation: 64.8 − 10.3 × (thermolabile MTHFR) − 5.31 × ( ≤ 1CRF). Screening of MTHFR polymorphisms may be useful in younger RVO patients, particularly when multiple CRF are absent.

## Introduction

Retinal vein occlusion (RVO) is the second most common retinal vascular disorder, after diabetic retinopathy, occurring mostly after the fourth decade of life^1,2^. The exact pathogenesis of RVO is unclear^3^. Cardiovascular systemic risk factors (CRF) such as the metabolic syndrome (arterial hypertension, hyperlipidemia and diabetes), stroke and smoking are common risk factors for RVO^3–6^. CRF are by far the most important risk factors for ‘typical’ RVO in patients older than 60, where most cases are found, since RVO prevalence increases with age^1,2^. Nevertheless, there are distinct clinical RVO sub-categories, with better visual outcome in younger patients^7^.

Data on associated thrombophilic risk factors (TRF) in various types of RVO have been published with reference to thrombophilia as a possible cause of RVO, mainly in those with a family history of thrombosis or aged under 60^8–10^.

Methylenetetrahydrofolate reductase (MTHFR) catalyzes the conversion of 5,10-methylenetetrahydrofolate into 5-methyltetrahydrofolate, which is the major circulating form of folate. Among other important functions, 5-methyltetrahydrofolate participates in the remethylation of homocysteine to methionine. Normal MTHFR activity may help  to maintain the pool of circulating folate and methionine, preventing a rise of homocysteine^11,12^. Hyperhomocysteinemia (HH) may be due to a genetic defect in the MTHFR, cystathionine synthase or methyltetrahydrofolate homocysteine methyltransferase enzymes, to an acquired nutritional deficiency in vitamin cofactors (folic acid, vitamins B6 and B12)^13^, to a chronic medical condition or to adverse drug effect^14,15^. HH has been related to atherogenesis, RVO and retinal artery occlusion^13,16^.

The C677T allele is characterized by a point mutation at position 677 of the MTHFR gene in chromosome 1 that converts a cytosine (C) into a thymine (T) in position 677 (677 C > T). This mutation results in an amino acid substitution (alanine to valine) in the enzyme. The result is a dysfunctional ‘thermolabile’ enzyme, so called because its activity is more than 60% lower at 37° among 677 C > T homozygotes (T677T), leading to HH^17^, while in heterozygotes such decreased activity is in an intermediate range. In the A1298C allele, the point mutation results in a substitution of glutamate for alanine in the enzyme, but homozygosity does not lead to HH, because the enzyme activity is reduced by 40%. However, combined heterozygosity, 667 C > T and 1298 A > C, leads to a similar profile of T677T homozygosity, resulting in HH that must be corrected by folate and vitamin B6 intake^11–13,15^.

The prevalence of MTHFR T677T genotype is 20% in China, 25% in Hispanics and 10–15% in North American Caucasians, while in Europe it is as low as 4% in Finland rising to 26% in southern Italy, being 11.8% in western Europe (Spain)^18^. C677T MTHFR heterozygosity prevalence is 6.6% in Africa, 26–37% in Japan, 24–40% in Europe (increasing from north to south) and 30–40% in American Caucasians^17,19^.

Although severe HH is rare, mild HH occurs in approximately 5% of the general population^15^. Mild HH is an independent risk factor for venous thromboembolism^12^. An association between HH, coronary heart disease, deep venous thrombosis and central retinal vein occlusion (CRVO) has been reported^16,20–22^.

Due to contradictory results, the role of MTHFR polymorphisms and HH in RVO is not well defined^23^. However, better understanding of associations between heritable thrombophilia and RVO is paramount, since thrombophilia may be associated with preventable/reversible deep vein thrombosis, pulmonary embolism, ischemic cerebral vascular accidents, osteonecrosis, sporadic and recurrent pregnancy loss and RVO of the fellow eye^12,20–22,24,25^.

Factor V G1691A (FV Leiden), factor II mutation or prothrombin (PT) gene mutation (PT G20210A), lupic anticoagulant, antiphospholipid antibody and plasminogen activator inhibitor (PAI-1), have been related with RVO^8^. PAI-1 mutations 4 G/5 G and 4 G/4 G are related with higher levels of PAI-1 which reduces activation of plasmin from plasminogen, reducing fibrinolysis.

The aim of the present study is to find whether RVO is associated with TRF and if age of RVO onset is an important factor to consider in the decision for screening such factors.

## Results

### Demographic characteristics

Sixty eyes of 60 white Caucasian patients (35 males, 25 females, p = 0.219) diagnosed with RVO were included. CRVO was present in 35 patients (58.3%) and BRVO in 25 patients (41.7%). Bilateral RVO was found in 6 patients (10.0%). Mean age at diagnosis was 64.0 ± 13.5. Males had lower mean age at diagnosis (62.1 ± 11.31 versus 68.5 ± 16.0 in females, p = 0.219, Table 1).

**Table 1:** Demographic characteristics of the RVO population.

n		RVO		Mean age at diagnosis (mean ± SD; age range)	
60 patients		Central	Branch	64.0 ± 13.5; 21–91	
Male	Female	58.3%	41.7%	Male	Female
58,3%	41.7%			62.1 ± 11.3; 38–85	66.5 ± 16.0; 21–91

n = number of patients, RVO = retinal vein occlusion, SD = standard deviation.

### Thrombophilic risk factors (TRF)

HH was found in 26 patients (43.3%, Table 2). Nineteen of these patients had CRVO (19 out of 32, 59.4% of the total CRVO cases) and 7 had BRVO (7 out of 22, 31.8% of the total BRVO cases). Males were 65.4% (n = 17) versus 34.6% females (n = 9, p = 0.377). Mean age at RVO diagnosis in HH patients (64.6 ± 13.7) was not significantly different from patients with normal homocysteine levels (63.1 ± 10.5, *p* = 0.658). PT mutation and FV Leiden mutation were found in one patient each. Lupic anticoagulant was found in 8.3% of the patients (Table 2). These patients were non-statistically significantly younger (Supplementary Table 1). PAI-1 mutations 4 G/4 G and 4 G/5 G were found in 40 patients (66.7%). There was no difference in age of RVO diagnosis between normal 5 G/5 G (64.7 ± 16.8) versus hypofibrinolytic 4 G/5 G heterozygosity (63.0 ± 12.3, p = 0.693)), 4 G/4 G homozygosity (67.0 ± 12.7, p = 0.744) or both 4 G/5 G and 4 G/4 G mutations [(p = 0.613), Anova F(2.55) = 0.263; p = 0.769)].

**Table 2:** TRF for RVO other than MTHFR polymorphisms.

HH	PT G20210A	Lupic anticoagulant	Antiphospholipid antibody	FV Leiden G1691A	PAI 1 4 G/4 G and 4 G/5 G	
	HTZ			HTZ	HOMO	HTZ
26 (43.3%)	1 (1.7%)	5 (8.3%)	0 (0%)	1 (1.7%)	7 (11.7%)	33 (55%)

TRF = thrombophilic risk factors; RVO = retinal vein occlusion; MTFRH = methylene tetrahydrofolate reductase; HH = Hyperhomocysteinemia (homocysteinemia > 15 µmol/L); FV Leiden = Factor V G1691A; PT G20210A = allele mutation of the factor II/prothombin gene; PAI-1 = plasminogen activator inhibitor-1 mutations 4 G/4 G and 4 G/5 G. HTZ = heterozygosity; HOMO = Homozygosity. Results are expressed in number of subjects and percentage.

MTHFR polymorphisms were found in 55 patients (91.7%). Homozygosity T677T was found in 9 patients (15.0%), heterozygosity C677T in 15 patients (25.0%) and more than 1 MTHFR polymorphism in 16 patients (26.7%, Table 3).

**Table 3:** MTHFR polymorphisms.

	CRVO	BRVO	Total RVO	Age (M ± SD)
T677T	4 (11.4%)	5 (20.0%)	9 (15.0%)	**52.3 ± 17.4**
C677T/A1298C	10 (8.6%)	6 (24.0%)	16 (26.7%)	**60.6 ± 13.3**
C1298C	1 (2.9%)	4 (16.0%)	5 (8.3%)	66.4 ± 10.5
C677T	12 (34.3%)	3 (12.0%)	15 (25.0%)	69.1 ± 8.30
A1298C	4 (11.4%)	6 (24.0%)	10 (16.7%)	67.1 ± 12.0
Total	**31 (88.6%)**	**24 (96.0%)**	**55 (91.6%)**	64.0 ± 13.5
No polymorphism	4 (11.4%)	1 (4.0%)	5 (8.3%)	71.6 ± 14.0

MTHFR = methylenetetrahydrofolate reductase; CRVO = central retinal vein occlusion; BRVO = branch retinal vein occlusion; age = age at RVO diagnosis; M = mean; SD = standard deviation.

MTHFR forms associated with decreased enzyme activity (T677T homozygosity or C677T/A1298C combined heterozygosity) were present in 41.7% of the patients (n = 25, out of 60). A total of 56.0% had CRVO (n = 14, out of 35, 44.0% of total CRVO patients) and 44.0% had BRVO (n = 11, out of 25, 40.0% of total BRVO patients).

Patients with MTHFR T677T homozygosity had the lowest mean age at diagnosis (52.3 ± 17.4, Table 3). Thermolabile MTHFR forms associated with decreased enzyme activity (T677T or C677T/A1298C) had a significant lower mean age [57.6 ± 15.1; t (58) = 3.32; p = 0.002; d = 0.846] when compared with patients in all other forms including the absence of polymorphisms (68.5 ± 10.2). Males were 64.0% (n = 16, out of 25, p = 0.452, Supplementary Fig. 1).

### Cardiovascular risk factors (CRF)

More than 1 CRF were present in 36 patients (60%). Arterial hypertension was present in 43 patients (71.7%) and hyperlipidemia in 30 patients (50.0%). Acute arterial events such as stroke or myocardial infarction were present in 7 patients (11.7%). A total of 21 patients (35.0%) were taking antiaggregants and 10 (16.7%) were taking anticoagulants. Previous history of more than one CRF was reported in 36 patients (60.0%), 23 with CRVO and 13 with BRVO. Males were 19 of such patients, (52.8%). Mean age at diagnosis of RVO was significantly higher in cases with >1 CRF (66.7 ± 12.9 versus 59.5 ± 13.7 with ≤1 CRF [*t*(57) = −2.05, *p* = 0.045, *d* = 0.54).

When considering CRF (>1 CRF *versus* ≤ 1 CRF) and thermolabile forms of MTHFR (T677T and C677T/A1298C), the mean age at diagnosis of RVO may be significantly predicted [F(2,56) = 7.18; *p* = 0.002] by the equation: 64.8–10.3 × (thermolabile MTHFR) − 5.31 × (≤1CRF).

For instance, a patient with ≤1 CRF and a thermolabile form of MTHFR would have a predicted mean age for a RVO event at age of 49 (49.2). On the other hand, a patient with >1 CRF and a non-thermolabile form of MTHFR would have a predicted mean age of diagnosis at age of 65 (64.8).

The difference would still be significant in case we include the C1298C genotype with the aforementioned two, as well [F(2,56) = 5.72; *p* = 0.005] by the equation: 69.5–9.04 × (thermolabile MTHFR + C1298C) −5.00× (≤1 CRF)].

## Discussion

The pathogenesis of RVO is not completely understood yet. The condition may be due to a combination of local or systemic factors known as the Virchow’s triad: (i) hemodynamic changes (venous stasis), (ii) degenerative changes of the vessel wall, and (iii) blood hypercoagulability^26^. Our study found a lower mean age at diagnosis in males. This may be related to the role of CRF, since retinal venous obstruction may be associated with significant cardiovascular morbidity^3,4,26^. We found that arterial hypertension and dyslipidemia were present in the majority (64.4%) of patients with >1 CRF. Patients with >1 CRF had a significantly higher mean age at diagnosis, as previously reported^27^. An extensive and expensive workup for thrombophilic disease may be useless in the vast majority of patients with RVO. Several reviews concluded that TRF screening is not cost-effective and should not be performed in all RVO cases^23,28,29^. However, TRF screening may be relevant in specific “atypical” cases, such as RVO in patients aged less than 60, in patients without CRF, when there is involvement of both eyes or when a family history of systemic thrombosis is present^22,24^. The results of our study agree with data previously published where TRF are particularly important in younger patients without known CRF^9,10,30^.

We found an increased prevalence of HH in patients with RVO. HH has been reported as an independent predictor factor for atherosclerosis and for thrombosis^31,32^. A review demonstrated that HH and anticardiolipin antibodies are associated with RVO^16^. The prevalence of MTHFR polymorphisms and the proportion of HH in normal patients varies among the different studies^12,17,19,33^. Some factors, like the ethnicity and geography, may be a bias when comparing the prevalence of MTHFR polymorphisms in different RVO studies. MTHFR T677T homozygosity was previously reported to be significantly more prevalent in patients with RVO^24^, including ischemic CRVO^32^. Thermolabile 677 C > T polymorphisms, such as T677T and compound heterozygous C677T/A1298C, along with 1298 A > C homozygosity, were found to be related with significant MTHFR enzyme decreased activity, mild HH or decreased folate levels^17,34–36^, including in large studies^37^. In our study MTHFR T677T was found in 11.7% of patients, which is in accordance with the prevalence of 11.8% found in the general population of nearby Spain^18^. However, MTHFR compound heterozygosity C677T/A1298C was found in 26.7% of patients, more than previously reported for the general population in Canada^34^, but less than in south-eastern Europe^38^. Homozygosity C1298C was found in 5% of patients which is a percentage in-between data previously described by the aforementioned groups^34,38^. More importantly, we found a significantly lower mean age at diagnosis in patients with the homozygous thermolabile form T677T. Furthermore, it was possible to build a model where lower mean age at diagnosis may be significantly predicted in the presence of MTHFR polymorphisms associated with decreased enzyme activity and <1 CRF. Overall, more than the relative prevalence of polymorphisms found in RVO, it is the association of thermolabile MTHFR polymorphisms and younger age at diagnosis that seems to be more relevant. This is important, because the ophthalmologist may be the first physician to identify patients suffering from thrombophilia and prone to systemic complications^39^, particularly in younger patients^8,10,40^. While we do realize that RVO is not a single defined disease, but a complex group of diseases where individualized and local factors play a role along with systemic and hematologic factors, search for TRF in the younger group may yield some clues about the patients at risk for recurrent RVO or complications of systemic arteriovenous occlusive disease^4,7^.

Our results partially agree with Russo *et al*. in that we did not found FV Leiden G1691A or PAI-1 (4 G/4 G and 4 G/5 G) to be correlated with RVO. Conversely, we did not find prothrombin (Factor II) gene mutation PT G20210A to be correlated with RVO, either^24,40^. The prevalence obtained for FV Leiden G1691A, PT G20210A and PAI-1 mutations 4 G/4 G or 4 G/5 G was similar to those reported for healthy people^8,24^ and age was not a differentiation factor between subgroups. However, the score obtained for PAI-1 mutations (64.7%) agrees with datum previously reported for RVO in a prospective work where it was found statistically significantly superior to a control group^40^. Our results do not agree with previous data indicating lupic anticoagulant and antiphospholipid antibody associated with RVO^8,16^.

This study has limitations. It is an institutional retrospective, consecutive case series report. We collected more data on TRF from CRVO than from BRVO patients’ files. This happened with other studies as well^40^. There are several possible explanations for this discrepancy with the incidence of either form of RVO^2^: (i) CRVO patients are more prone to be referred from the emergency department to the medical retina department, where the searching for TRF is based on, due to its worse prognosis and increased treatment burden^2^, (ii) due to the former reason, CRVO patients are more prone to stay for longer at the hospital visiting scheduling, increasing the outpatient hospital relation CRVO/BRVO when comparing with the general population, (iii) systematic search for TRF began with CRVO cases in our department. It is possible that the results of this study cannot be universally extrapolated due to the aforementioned geographic variations of MTHFR polymorphisms and to the geographic variation of HH^41^. Nevertheless, we have made an effort to compare our results with the results of other studies, including studies on the general healthy population of our own area. The size of our sample of 60 RVO cases is relatively small, due to the interruption of systematic search for MTHFR polymorphisms in RVO patients in our department, based on costs and on disputable usefulness for its use. The strengths of this study are related to age stratification of the results and the comparison with data from RVO studies and studies on healthy population of different geographic areas, including our own.

To conclude, it seems important to consider searching for TRF, including MTHFR polymorphisms in ‘atypical’ RVO patients, younger at diagnosis or lacking multiple CRF.

## Methods

Retrospective institutional consecutive case series of RVO patients (CRVO and BRVO) admitted between 2013 and 2018. CRVO and BRVO were diagnosed by characteristic fundus features. CRF, such as arterial hypertension, hyperlipidemia, atrial fibrillation and diabetes, were collected, as well as acute cardiovascular and cerebrovascular events. The exclusion criteria included renal disease, cancer and medication with supplements or drugs acting on homocysteine levels. TRF screening was performed using the CVD-StripAssay (ViennaLab Diagnostics GmbH, Vienna, Austria). Briefly, genomic DNA was extracted from whole blood; the different gene sequences were simultaneously amplified using a multiplex polymerase chain reaction (PCR) with biotin-labeling of the products; the products were hybridized on StripAssay® test strips and then detected by streptavidin-alkaline phosphatase. Total L-homocysteine was quantified in serum using the ARCHITECT one-step immunoassay (Abbott Diagnostics, Abbott Laboratories, Abbott Park, IL, USA) with Chemiluminescent Microparticle Immuno Assay (CMIA) technology. The results of TRF screening were collected from data files: MTHFR 677 C > T and 1298 A > C polymorphisms, factor V1691A (FV Leiden), PT 20210 A, PAI-1, HH (homocysteine level > 15 µmol/L)^15^. The study follows the Declaration of Helsinki and was approved by the local ethics committee of the Leiria Hospital Center. All participants provided informed consent. Data analysis was performed with “Statistical Package for the Social Sciences-IBM SPSS Statistics24®”. Two-tailed *p*-values < 0.05 [Confidence Interval (CI) 95%] were considered to be statistically significant. After testing for normality with the Shapiro-Wilk test, one-way ANOVA or independent sample t-test were performed. Two different effect size measures were calculated: *partial eta squared* (η_p_^2^) [small 0.01, medium 0.06, large 0.14] and *Cohen’s d* scores [small 0.2, medium 0.5, large 0.8].

## Supplementary information

Supplementary Dataset 1

Supplementary Dataset 2
