# Supplementary material for: Thrombophilic risk factors for retinal vein occlusion
Source: Sci Rep. 2019 Dec 12;9:18972. doi: 10.1038/s41598-019-55456-5 (PMC6908668; doi:10.1038/s41598-019-55456-5)
Supplement: Supplementary file 1 — Supplementary Dataset 1 [file 41598_2019_55456_MOESM1_ESM.docx]

**Thrombophilic risk factors for retinal vein occlusion**

Maria J. Vieira * (1), António Campos (1,2,3), Anália do Carmo (4,5), Henrique Arruda (1),

Joana Martins (1), João P Sousa (1,3,6)

1Ophthalmology Department, Centro Hospitalar de Leiria, Leiria, Portugal

2Coimbra Institute for Clinical and Biomedical Research (iCBR), Faculty of Medicine,

University of Coimbra, Coimbra, Portugal

3ciTechCare, Center for Innovative Care and Health Technology, Instituto Politécnico

de Leiria, Leiria, Portugal

4Clinical Pathology Department, Centro Hospitalar Universitário de Coimbra (CHUC),

Coimbra, Portugal

5CNC.iCBR Consortium, University of Coimbra, Coimbra, Portugal

6Medical Sciences Department, Faculty of Health Sciences, University of Beira Interior,

Covilhã, Portugal


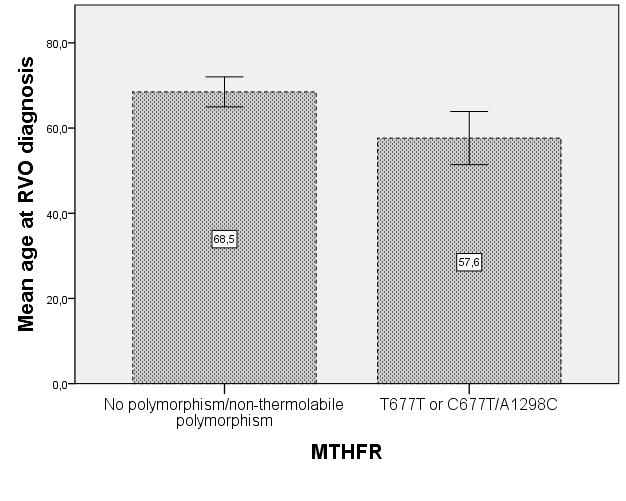


**Supplementary Figure 1: Mean age at RVO diagnosis among MTHFR**

**thermolabile form**, with a significantly lower mean age at diagnosis [t(39.2) =2.79;

*p*=.008; d=0.753].
